# OLDER WORKERS’ DEMOGRAPHIC AND SOCIOECONOMIC CHARACTERISTICS BY DIGITAL SKILL USE PATTERNS IN THE UNITED STATES

**DOI:** 10.1093/geroni/igae098.3103

**Published:** 2024-12-31

**Authors:** Takashi Yamashita, Donnette Narine, Runcie Chidebe, Phyllis Cummins, Jenna Kramer, Rita Karam

**Affiliations:** University of Maryland Baltimore County, Baltimore, Maryland, United States; University of Maryland Baltimore County, Baltimore, Maryland, United States; Miami University, Oxford, Ohio, United States; Miami University, Oxford, Ohio, United States; RAND Corporation, Arlington, Virginia, United States; RAND Corporation, Santa Monica, California, United States

## Abstract

In the current technology-rich society, having sufficient skills to use information and communication technology (ICT) for problem-solving is advantageous in the labor market from a human capital perspective. However, older workers tend to have lower digital problem-solving skills than their younger counterparts. Practice engagement theory suggests that continuous use of skills can promote and maintain skill proficiency over the life course. The current study examined the digital problem-solving skills use patterns among older workers in the United States. Nationally representative samples of workers aged 50 years and older (n = 1,700) were obtained from the 2012/2014/2017 U.S. Program for the International Assessment of Adult Competencies (PIAAC) restricted-use-file pooled data. Latent class analysis with seven ICT use indicators, such as email, spreadsheet, and virtual discussions, at home and work (14 in total) identified two distinctive underlying subgroups, including ubiquitous ICT users and infrequent ICT users. The ubiquitous ICT users had a greater average digital problem-solving skills score (280 out of 500 points) than infrequent ICT users (244 points). About 25% of the ubiquitous users used virtual discussions at work, while only 3% of the infrequent users did. Subsequent binary logistic regression showed that the ubiquitous users were more likely to be younger, women, college-educated, and higher income earners than the infrequent users. Findings are useful to identify older workers who may be at risk of being digitally left behind in the labor market, as well as to inform digital problem-solving skills promotion interventions based on the ubiquitous users’ characteristics.

